# Real‐world efficacy and toxicity of ipilimumab and nivolumab as a first‐line treatment for advanced renal cell carcinoma according to IMDC risk criteria—A multi‐center retrospective analysis on behalf of the GUARDIANS group

**DOI:** 10.1002/ijc.70267

**Published:** 2025-11-29

**Authors:** Hendrik Dinkel, Linus Materna, Ramona Stelmach, Stefanie Zschäbitz, Stephanie Neuberger, Can D. Aydogdu, Jozefina Casuscelli, Timo Egenolf, Matteo Silberg, Julie Steinestel, Arne Strauss, Florian Kirchhoff, Marit Ahrens, Pia Paffenholz, Richard Cathomas, Berna C. Özdemir, Christopher Gossler, Philipp Ivanyi, Marc Rehlinghaus, Thomas Hilser, Viktor Grünwald, Katrin Schlack

**Affiliations:** ^1^ Department of Urology University Hospital Münster Münster Germany; ^2^ Department of Medical Oncology, National Center for Tumor Diseases Heidelberg University Hospital Heidelberg Germany; ^3^ Interdisciplinary Working Group Kidney Cancer of the German Cancer Society (IAGN‐DKG); ^4^ Department of Urology and Pediatric Urology University Medical Center of Johannes Gutenberg University Mainz Germany; ^5^ Department of Urology LMU University Hospital, Ludwig Maximilian University of Munich Munich Germany; ^6^ Department of Urology and Pediatric Urology University Hospital Würzburg Würzburg Germany; ^7^ Department of Urology Marienhospital Herne, Ruhr University Bochum Herne Germany; ^8^ Department of Urology University Hospital Augsburg Augsburg Germany; ^9^ Department of Urology University Medicine Göttingen Göttingen Germany; ^10^ Department of Urology TUM University Hospital Rechts der Isar Technical University of Munich Munich Germany; ^11^ Department of Oncology University Hospital Frankfurt Frankfurt Germany; ^12^ Department of Urology, Uro‐Oncology, Robot Assisted and Reconstructive Urologic Surgery University of Cologne Faculty of Medicine and University Hospital Cologne Cologne Germany; ^13^ Division of Oncology/Hematology Kantonspital Graubünden Chur Switzerland; ^14^ Department of Medical Oncology Inselspital Bern Bern Switzerland; ^15^ Department of Urology, Krankenhaus St. Josef University of Regensburg Regensburg Germany; ^16^ Department of Hematology, Hemostasis, Oncology, and Stem Cell Transplantation, Claudia‐von Schelling Comprehensive Cancer Center Hannover Medical School Hannover Germany; ^17^ Department of Urology University Hospital and Medical Faculty, Heinrich‐Heine‐University Düsseldorf Düsseldorf Germany; ^18^ Centre for Integrated Oncology (CIO) Düsseldorf CIO Aachen‐Bonn‐Cologne‐Düsseldorf Düsseldorf Germany; ^19^ Department of Internal Medicine (Tumor Research) University of Duisburg‐Essen, German Cancer Consortium (DKTK)‐University Hospital Essen Essen Germany; ^20^ Department of Urooncology University of Duisburg‐Essen, German Cancer Consortium (DKTK)‐University Hospital Essen Essen Germany

**Keywords:** immunotherapy, ipilimumab, nivolumab, real‐world, renal cell carcinoma

## Abstract

Ipilimumab and nivolumab are recommended as first‐line therapy for patients with metastatic or advanced renal cell carcinoma (aRCC) and International Metastatic RCC Database Consortium (IMDC) intermediate or poor risk. We retrospectively evaluated efficacy and safety in a multi‐center real‐world cohort with 356 patients initiating ipilimumab and nivolumab from 17 centers in Germany and Switzerland. Median age was 64 years, most patients were male (69.1%) and had clear cell histology (74.1%). IMDC risk was intermediate in 61.8% and poor in 28.7%. About 37.1% of cases did not meet the inclusion criteria for the CheckMate 214 pivotal study (e.g., poor Eastern Cooperative Oncology Group [Performance Status Scale] [ECOG] status, comorbidities, brain metastases, and impaired renal function). After a median follow‐up of 17.5 months, complete response was seen in 8.7%, partial response in 28.7% of patients. Median progression‐free survival (PFS) was 8 (95% confidence interval [CI] 5.4–10.6) and median overall survival (OS) 39 months (95% CI 27.5–50.5). Subgroup analysis of patients with non‐clear cell histology showed a shorter PFS and OS. Other negative predictors were poor ECOG, fewer induction cycles, ineligibility to pivotal study, and hepatic metastases. Adverse events occurred in 76.4% of patients (35.4% ≥ grade 3). High‐dose corticosteroids were applied in 27.3% of cases. Cabozantinib was most frequently administered (63.4%) as subsequent therapy and showed superior OS and PFS compared to other second‐line options. Our data support ipilimumab and nivolumab as a first‐line treatment of aRCC with robust efficacy and safety. Patient selection was less restrictive in our clinical practice and may explain differences to CheckMate 214 trial.

## Graphical abstract

What's new?

The combination of ipilimumab and nivolumab as a first‐line treatment for advanced renal cell carcinoma has demonstrated durable response and tolerability in clinical trials. However, inclusion criteria for clinical studies are restrictive, and patients in the real‐world setting tend to have more comorbidities. Here, the authors retrospectively evaluated efficacy and safety in a multi‐center real‐world cohort of 356 patients initiating ipilimumab and nivolumab from 17 centers in Germany and Switzerland. The results support the efficacy and safety of ipilimumab and nivolumab combination in routine clinical practice, even with patients being frailer than those in the pivotal trial.

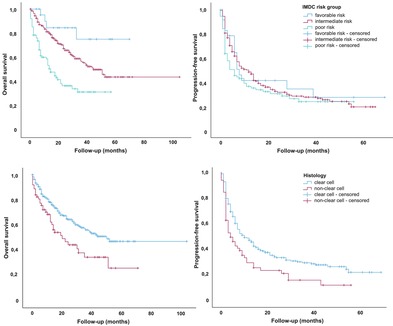

**Abbreviations**
AE: adverse event
ALT: alanine aminotransferase
aRCC: advanced renal cell carcinoma
ASCO: American Society of Clinical Oncology
AST: aspartate aminotransferase
ccRCC: clear cell renal cell carcinoma
CI: confidence interval
CR: complete response
CTCAE: Common Terminology Criteria for Adverse Events
CTLA‐4: cytotoxic T‐lymphocyte antigen 4
DCR: disease control rate
DoR: duration of response
ECOG: Eastern Cooperative Oncology Group (Performance Status Scale)
ESMO: European Society for Medical Oncology
HR: hazard ratio
IMDC: International Metastatic RCC Database Consortium
IO: immunooncology
IQR: interquartile range
ITT: intention‐to‐treat
KMA: Kaplan–Meier analysis
ORR: objective response rate
OS: overall survival
PD‐1: programmed cell death protein 1
PFS: progression‐free survival
PR: partial response
RCC: renal cell carcinoma
RECIST: Response Evaluation Criteria in Solid Tumors
TKI: tyrosine kinase inhibitor
VEGF: vascular endothelial growth factor

## 1 INTRODUCTION

Renal cell carcinoma (RCC) is one of the most common cancers worldwide affecting mostly men.^1^ Most frequently the tumor is diagnosed incidentally by ultrasound at early disease stages and can be cured by surgical excision after confirmation in Computed Tomography (CT)‐scan or Magnetic Resonance Imaging (MRI). In some cases though, the tumor progresses unnoticed and these patients initially present with an advanced or metastatic disease.^2^

The treatment of advanced and metastatic renal cell carcinoma (aRCC) beyond surgery has long been a challenge due to the inherent resistance of RCC to chemotherapy and radiation. The implementation of tyrosine kinase inhibitors (TKI) and later immune checkpoint inhibitors (immunooncology [IO]) has drastically changed the therapeutic landscape over the past two decades. Especially combination therapies have shown benefit in clinical trials in terms of efficacy as well as reduced toxicity and therefore have secured their place as first‐line treatment option. However, increased toxicity has also been associated with more intensive treatment regimens. Patients with aRCC are categorized in three risk groups (good, intermediate, and poor prognosis) according to International Metastatic Renal‐Cell Carcinoma Database Consortium (IMDC) risk criteria; ipilimumab and nivolumab are only approved for the intermediate and poor risk group.^2^

At present four different combination therapies are recommended in the first‐line setting, in particular IO/IO‐ or IO/TKI‐regimens. Ipilimumab (IO, cytotoxic T‐lymphocyte antigen 4 [CTLA‐4] directed antibody) and nivolumab (IO, programmed cell death protein 1 [PD‐1] directed antibody) (CheckMate 214 study) is the only approved IO/IO‐combination. Available IO/TKI‐combination therapies are pembrolizumab (IO, PD‐1) with lenvatinib (TKI, multi‐kinase inhibitor) (CLEAR study, NCT02811861), nivolumab, and cabozantinib (TKI, vascular endothelial growth factor [VEGF]‐inhibitor) (CheckMate 9ER study, NCT03141177) as well as pembrolizumab and axitinib (TKI, multi‐kinase inhibitor) (KEYNOTE 426 study, NCT02853331).^3^, ^4^, ^5^ Combination of avelumab (IO, antibody directed to ligand of PD‐1 [PD‐L1]) with axitinib has not shown significant overall survival (OS) benefit over sunitinib in JAVELIN Renal 101 (NCT02684006) study and is not recommended in current guidelines.^6^, ^7^ Alternative therapies for patients unfit for one of the above mentioned combination treatments are TKI monotherapies (sunitinib or pazopanib for all risk groups or cabozantinib for IMDC intermediate and poor risk patients).^6^

Ipilimumab is an anti‐cytotoxic T‐lymphocyte‐associated antigen‐4 antibody (CTLA‐4), blocking the negative regulatory activity of tumor cells against T‐cell immune response.^8^ Its efficacy was first shown in patients with metastatic melanoma.^9^, ^10^

Nivolumab is a programmed death‐1 (PD‐1) immune checkpoint inhibitor, also blocking the escape mechanism of the tumor cells from T‐cell mediated killing.^11^ In a large comparative study, the combination of ipilimumab and nivolumab showed advantageous response rates in metastatic melanoma when compared with the respective monotherapy.^12^ The combination therapy is given for the first four cycles with nivolumab 3 mg/kg plus ipilimumab 1 mg/kg every 3 weeks, followed by a monotherapy with nivolumab 240 mg every 2 weeks or 480 mg every 4 weeks.^13^

The phase III, open‐label, randomized CheckMate 214 trial showed a benefit in OS, progression‐free survival (PFS), duration of response (DoR) and objective response rate (ORR) alongside manageable rates of adverse events (AEs) for ipilimumab and nivolumab compared to sunitinib alone. These findings were underlined by the final 9‐year follow‐up data presented at American Society of Clinical Oncology (ASCO) 2025 showing similar results to the pivotal trial: median OS (mOS) was 52.7 versus 37.8 months for ipilimumab and nivolumab versus sunitinib, OS probabilities were 31% versus 20%, ORR was 39.5% versus 33% all for the entire population. Ipilimumab and nivolumab confers a significant benefit in terms of its very long median DoR with 76.2 months.^14^, ^15^

In terms of AEs, nearly all patients showed treatment‐related AE (94% under ipilimumab and nivolumab). Most frequent AEs were fatigue, pruritus, and rash, diarrhea and nausea. Grades 3 and 4 toxicities occurred in 49% of the patients with lipase‐elevation, fatigue, and diarrhea being the most common. Therapy discontinuation was necessary in 24% under ipilimumab and nivolumab and 21% of the patients starting ipilimumab and nivolumab did not receive all four doses of the initiation therapy.^15^, ^16^

Due to the fact that inclusion criteria for clinical studies are restrictive, patients in the real‐world setting tend to have more comorbidities and often a worse performance status. These patients are therefore not represented in clinical trials but still need to be treated. To examine whether the administration of ipilimumab and nivolumab is efficacious and safe in routine clinical practice, we collected and analyzed data from a real‐world cohort of patients.

## 2 MATERIALS AND METHODS

### 2.1 Patients

We retrospectively collected data from 356 patients initiating therapy with ipilimumab and nivolumab for aRCC in 17 clinical centers in Germany and Switzerland between 2015 and 2023. The data encompassed baseline demographic and clinical characteristics.

Ipilimumab was given with a dose of 1 mg/kg body weight along with 3 mg/kg nivolumab every 3 weeks for the first four cycles, followed by nivolumab monotherapy with a dose of 240 mg every 2 weeks or 480 mg every 4 weeks.

### 2.2 Survival analysis and statistical methods

Statistical analyses were performed using SPSS version 29 (IBM Inc., Armonk, NY). Follow‐up was calculated in months from initiation of ipilimumab and nivolumab or second‐line therapy to time of death or last follow‐up known. Tumor response was assessed by imaging with CT and/or MRI and defined based on local standards in each center and according to Response Evaluation Criteria in Solid Tumors (RECIST) 1.1. A central radiological post hoc review was not performed.

PFS, OS, and DoR were estimated using the Kaplan–Meier analyzes (KMA). PFS was measured as the time from initiation of therapy to radiological or clinical disease progression or death by any cause, whatever occurred first. OS was defined as the time of therapy initiation to death by any cause. DoR was specified as the time from first therapy response (complete response [CR] or partial response [PR]) to disease progression or death, whatever occurred first. The ORR was defined as the proportion of patients achieving CR or PR; disease control rate (DCR) was the proportion of patients with CR, PR, or stable disease (SD) for at least 12 weeks. Medians were reported with interquartile ranges (IQR) or 95% confidence intervals (CI).

For uni‐ and multivariate analysis the Cox proportional‐hazards regression model was used. The hazard ratio (HR) was reported with 95% CI. For statistical analysis of differences between groups, the Log‐rank test was performed and a *p*‐value <.05 was considered statistically significant. For KMA and Cox regression, patients without any event of disease progression or death were censored.

We collected information on AEs and analyzed the data descriptively. AEs were recorded according to Common Terminology Criteria for Adverse Events (CTCAE) version 5.0.

## 3 RESULTS

### 3.1 Baseline characteristics

A total of 356 patients were included with a median age of 64 years (IQR 55–71). 69.1% of the patients were male and ECOG performance status was ≥2 in 14.3%. 61.8% were in the IMDC intermediate and 28.7% in the poor risk group. About 37.1% of patients were not included in the CheckMate 214 trial due to poor ECOG, uncontrolled brain metastases and comorbidities such as autoimmune diseases and impaired renal function. In 74.1% of the patients, the histology showed a clear cell renal cell carcinoma (ccRCC) or predominant clear cell component, 8.1% had a papillary RCC, 7.3% predominant sarcomatoid differentiated RCC and 2.2% showed chromophobe histology; the remaining 8.2% of patients had rare subtypes (e.g., with PIK3CA‐mutated), were not otherwise specified or had unknown histology. About 75.6% of patients had metastases at more than one site at therapy initiation (Table 1).

**TABLE 1:** Baseline characteristics of advanced renal cell carcinoma patients treated with ipilimumab and nivolumab in a real‐world setting.

Characteristic	All	Favorable risk	Intermediate risk	Poor risk	Not classified
Patients—no. (%)	356 (100)	27 (7.6)	220 (61.8)	102 (28.7)	7 (1.9)
Sex—no. (%)					
Male	246 (69.1)	20 (74.1)	158 (71.8)	62 (60.8)	6 (85.7)
Female	110 (30.9)	7 (25.9)	62 (28.2)	40 (39.2)	1 (14.3)
ECOG PS—no. (%)					
0	187 (52.5)	22 (81.5)	128 (58.2)	32 (31.4)	7 (100)
1	117 (32.9)	4 (14.8)	76 (34.5)	36 (35.3)	0
2	30 (8.4)	1 (3.7)	10 (4.5)	19 (18.6)	0
>2	21 (5.9)	0	6 (2.8)	15 (14.7)	0
Median age—years (IQR)	64 (55–71)	62 (51–68)	65 (55–72)	65 (55–72)	57 (26–63)
Prior nephrectomy—no. (%)	240 (67.4)	24 (88.9)	157 (71.4)	53 (52)	5 (71.4)
Clear cell histology—no. (%)	264 (74.1)	22 (81.5)	180 (81.8)	67 (65.7)	3 (42.9)
Most common sites of metastases—no. (%)					
Lung	244 (68.3)	15 (55.6)	149 (67.7)	77 (75.5)	4 (57.1)
Lymph nodes	189 (52.8)	13 (48.1)	111 (50.5)	61 (59.8)	4 (57.1)
Bone	128 (35.7)	7 (25.9)	72 (32.7)	48 (47.1)	2 (28.6)
Liver	74 (20.5)	5 (18.5)	34 (15.5)	33 (32.4)	2 (28.6)
Adrenal glands	50 (14)	2 (7.4)	31 (14.1)	15 (14.7)	2 (28.6)
Brain	30 (8.4)	2 (7.4)	17 (7.7)	12 (11.8)	0
No. of sites—no. (%)					
1	87 (24.4)	6 (22.2)	65 (29.5)	12 (11.8)	2 (28.6)
≥2	269 (75.6)	21 (77.8)	153 (69.6)	90 (88.2)	5 (71.4)

Abbreviations: ECOG PS, Eastern Cooperative Oncology Group Performance Status; IQR, interquartile range; No., number.

Median follow‐up time was 17.5 months (IQR 7–34). About 55.3% received all four cycles of initiation therapy with ipilimumab and nivolumab. About 16.3% of the patients were still on treatment with nivolumab at the time of evaluation. Reasons for discontinuation of nivolumab were mostly disease progression (57.2%) and toxicity (26.5%).

### 3.2 Clinical efficacy of nivolumab and ipilimumab

**FIGURE 1:**
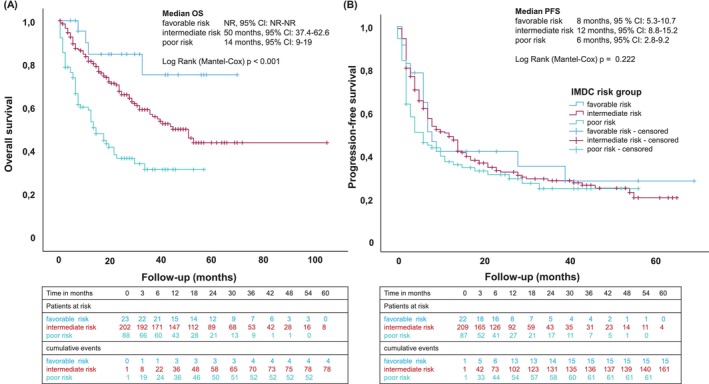
Kaplan–Meier estimates of overall survival (OS) (A) and progression‐free survival (B) in a real‐world cohort of advanced renal cell carcinoma patients treated with ipilimumab and nivolumab, stratified by International Metastatic Renal‐Cell Carcinoma Database Consortium Score (IMDC) risk categories (favorable, intermediate and poor). CI, confidence interval; NR, not reached.

In terms of the efficacy, ORR was 37.4% with CR rate in 8.7% and PR in 28.7% of patients. SD was observed in 19.9% resulting in a DCR of 57.3%. Mixed response was observed in 5.3% of patients. Forty‐seven patients died or were lost to follow‐up before the first staging. The median PFS (mPFS) in our cohort was 8 months (95% CI 5.4–10.6) and median OS was 39 months (95% CI 27.5–50.5). In the intermediate risk group median PFS was 12 months (95% CI 8.8–15.2) and median OS 50 months (95% CI 37.4–62.6). For poor risk patients, a median PFS of 6 months (95% CI 2.8–9.2) and median OS of 14 months (95% CI 9–19) was perceived (Figure 1, Table 2). In patients with CR the median DoR was not reached (NR) at time of data collection and with PR the median DoR was 18 months (95% CI 9.4–26.6).

**TABLE 2:** Outcome of advanced renal cell carcinoma (RCC) patients treated with ipilimumab and nivolumab according to different International Metastatic RCC Database Consortium risk groups.

Variable	All	Favorable risk	Intermediate risk	Poor risk	Not classified
Median follow‐up—months (IQR)	17.5 (7–34)	25 (9.25–43)	19 (10–37.75)	12 (3–24)	43 (13–51)
Best clinical outcome—no. (%)					
CR	31 (8.7)	2 (7.4)	24 (10.9)	5 (4.9)	0 (0)
PR	102 (28.7)	6 (22.2)	65 (29.5)	30 (29.4)	1 (12.5)
SD	71 (19.9)	9 (33.3)	50 (22.7)	11 (10.8)	1 (12.5)
PD	86 (24.2)	6 (22.2)	53 (24.1)	25 (24.5)	2 (25)
MR	19 (5.3)	2 (7.4)	9 (4.1)	6 (5.9)	2 (25)
n/a	47 (13.2)				
ORR—no. (%)	133 (37.4)	8 (29.6)	89 (40.4)	35 (34.3)	1 (12.5)
DCR—no. (%)	204 (57.3)	17 (62.9)	139 (63.1)	46 (45.1)	2 (25)
Median PFS—months (95% CI)	8 (5.4–10.6)	8 (5.3–10.7)	12 (8.8–15.2)	6 (2.8–9.2)	NR (NR‐NR)
Median OS—months (95% CI)	39 (27.5–50.5)	NR (NR‐NR)	50 (37.4–62.6)	14 (9–19)	NR (NR‐NR)
Patients died—no. (%)	140 (39.3)	5 (18.5)	78 (35.5)	54 (52.9)	3 (37.5)

Abbreviations: CR, complete response; DCR, disease control rate; IQR, interquartile range; MR, mixed response; No., number; NR, not reached; ORR, objective response rate; OS, overall survival; PD, progressive disease; PFS, progression‐free survival; PR, partial response; SD, stable disease.

**FIGURE 2:**
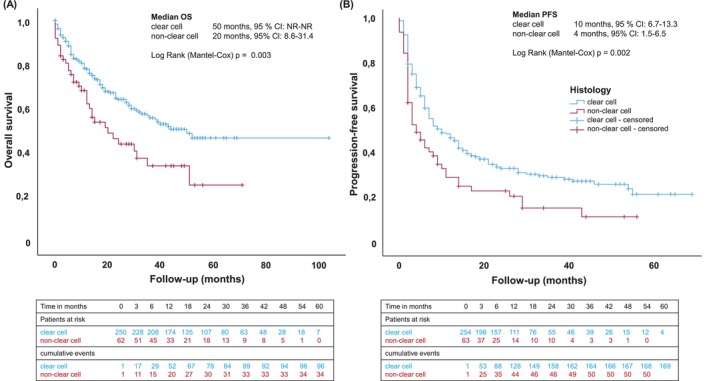
Kaplan–Meier estimates of overall survival (OS) (A) and progression‐free survival (PFS) (B) in a real‐world cohort of advanced renal cell carcinoma (RCC) patients treated with ipilimumab and nivolumab, stratified by histologic subtype (clear cell vs. non‐clear cell RCC). CI, confidence interval; NR, not reached.

Considering only the ccRCC patients, median DoR was slightly higher at 21 months (95% CI 0–43.9) after PR. In this group, median OS was 50 months (95% CI NR‐NR) and median PFS 10 months (95% CI 6.7–13.3). Patients with non‐ccRCC had a median OS of 20 months (95% CI 8.6–31.4, *p* = .003), median PFS of 4 months (95% CI 1.5–6.5, *p* = .002) and an ORR of 31.9% (Figure 2). Individuals with papillary histology showed a median OS of 20 months (95% CI 0.0–43.5) and median PFS of 5 months (95% CI 1.8–8.2), with predominant sarcomatoid differentiation a median OS of 31 months (95% CI NR‐NR) and median PFS of 14 months (95% CI 5.9–22.1) and a small cohort of chromophobe carcinomas had a median OS of 14 months (95% CI 8.9–19.1) and median PFS of 3 months (95% CI 1.7–4.3) (Table S1).

Patients from our cohort who would not have been included in the CheckMate 214 study according to the inclusion criteria showed a median OS of 23 months (95% CI 11.5–34.5), median PFS of 9 months (95% CI 4.9–13), and ORR of 29.3%. For patients with poor ECOG median OS was 8 months (95% CI 3.8–12.2) and median PFS 6 months (95% CI 0.1–11.9); in cases with autoimmune diseases median OS was 35 months (95% CI 8.3–61.7) and median PFS 12 months (95% CI 7–17); patients with impaired renal function (Glomerular Filtration Rate [GFR] <60 mL/min) showed a median OS of 50 months (95% CI 30.3–69.7) and median PFS of 10 months (95% CI 4.9–15.1) and median OS in the cohort with brain metastases was 17 months (95% CI 11–23) with a median PFS of 10 months (95% CI 6.7–13.3). The median OS of CheckMate 214 eligible patients in our cohort was NR; median PFS of this cohort was 8 months (95% CI 4.5–11.5) and ORR 42.1%.

Patients who received four cycles of ipilimumab and nivolumab in the beginning of their therapy showed significantly longer median OS (52 months, 95% CI NR‐NR) and median PFS (14 months, 95% CI 10.4–17.6) then those, who received one (mOS 13 months, 95% CI 2.3–23.7; mPFS 4 months, 95% CI 1.8–6.2), two (mOS 33 months, 95% CI 13.5–52.5; mPFS 5 months, 95% CI 1.2–8.8) or three (mOS 30 months, 95% CI NR‐NR; mPFS 4 months, 95% CI 1.2–6.8) initiation cycles (Figure S1).

**FIGURE 3:**
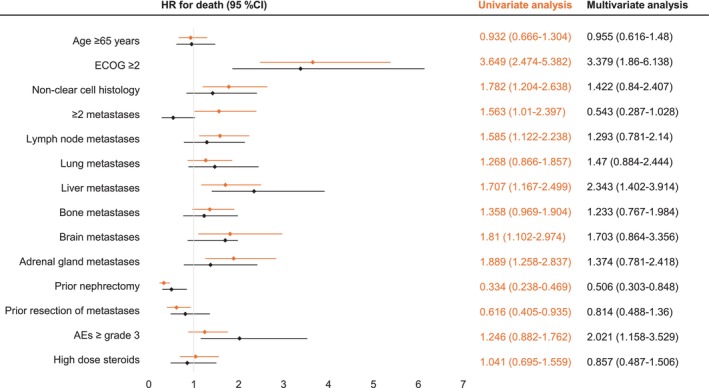
Forest plot of multi‐ and univariate analysis of median overall survival (OS) of advanced renal cell carcinoma patients treated with ipilimumab and nivolumab. AE, adverse event; CI, confidence interval; ECOG, Eastern Cooperative Oncology Group Performance Status; HR, hazard ratio.

Differences in OS between clinical groups were assessed using uni‐ and multivariate analyses. Univariate analysis showed shorter median OS in patients with poor ECOG, non‐clear cell histology as well as lymphonodal, hepatic, brain, and adrenal as well as ≥two metastases. Statistical significant longer median OS was detected in patients with prior nephrectomy or resection of metastases. Taking all variables into account, the multivariate analysis revealed a survival disadvantage for poor ECOG, liver metastases, and high‐grade AEs (Figure 3, Table S2). Shorter median PFS was observed in older patients (>65 years), non‐clear cell histology, and lymphonodal or hepatic metastases (Table S3).

### 3.3 Toxicity

Concerning the safety profile, we found that treatment‐related AEs occurred in 76.4% of the patients. AEs ≥ Grade 3 were documented in 35.4% of patients. These were most commonly fatigue (22.7%; ≥ Grade 3 2.2%), elevated liver enzymes or immune‐related hepatitis (19.1%; ≥ Grade 3 9%), rash (18.5%; ≥ Grade 3 2%), pruritus (18%; ≥ Grade 3 0.3%), thyroiditis (16.6%; ≥ Grade 3 2.5%), and diarrhea (14.9%; ≥ Grade 3 4.5%). About 24.7% of patients had to interrupt their therapy with ipilimumab and 16% with nivolumab due to AEs. Corticosteroids had to be applied in 37.1% with high dosage (≥40 mg/day prednisone) in 27.3% of the patients (Table 3).

**TABLE 3:** Treatment‐related adverse events and corticosteroid treatment in patients under ipilimumab and nivolumab.

Adverse events—no. (%)	Any grade	≥ Grade 3
Any	272 (76.4)	126 (35.4)
Fatigue	81 (22.7)	8 (2.2)
Hepatitis/elevated liver enzymes	68 (19.1)	32 (9)
Rash	66 (18.5)	7 (2)
Pruritus	64 (18)	1 (0.3)
Thyreoiditis	59 (16.6)	9 (2.5)
Diarrhea	53 (14.9)	16 (4.5)
Pneumonitis	34 (9.6)	11 (3.1)
Nausea	32 (9)	5 (1.4)
Arthralgia	25 (7)	8 (2.2)
Weight loss	24 (6.7)	2 (0.6)
Hypophysitis	21 (5.9)	9 (2.5)
Myalgia	18 (5.1)	3 (0.8)
Nephritis	17 (4.8)	6 (1.7)
Pancreatitis	14 (3.9)	2 (0.6)
Colitis	6 (1.7)	2 (0.6)
Corticosteroids—no. (%)	132 (37.1)	
High dose	97 (27.2)	

Abbreviation: No., number.

Multivariate analysis showed a significant higher risk of death in patients with AEs ≥Grade 3 (HR 2.021, 95% CI 1.158–3.529, *p* = .013). In addition, the ORR in this group was relevantly lower at 21.3% (CR 2.5%, PR 18.8%) than in patients without AEs ≥grade 3 (37.3%, CR 9.3%, PR 28%). Concerning median OS and PFS, Kaplan–Meier demonstrated comparable results in groups with any AE or AEs ≥ Grade 3, respectively (Table S4). ORR was similar between patients with any AE (42.7%) or without (38.1%). Patients who received high dosed corticosteroids did not have impaired median OS (HR 1.041, 95% CI 0.695–1.559), median PFS (HR 0.845, 95% CI 0.613–1.165) oder ORR (42.3% vs. 38.4%).

### 3.4 Clinical efficacy and outcomes of second‐line treatment

A subsequent therapy was administered in 182 cases (51.1%). For all patients receiving second‐line therapy, the median OS was 24 months (95% CI 18.7–29.7) and median PFS 11 months (95% CI 7.3–14.7) after initiation of second‐line therapy. Cabozantinib was the second‐line therapy most frequently applied (63.4%). Besides, several other second‐line options were administered, including sunitinib (11.3%), axitinib, pazopanib (both 4.3%), lenvatinib plus everolimus (3.2%), tivozanib (2.2%), and different IO/TKI combinations or study medication (e.g., belzutifan).

About 59.4% of patients receiving cabozantinib had progressed until last follow‐up. Median OS was 38 months (95% CI 20.7–55.3) and median PFS 15 months (95% CI 8.2–21.9) with cabozantinib in second‐line setting compared to a median OS of 16 months (95% CI 10.1–21.9, *p* = .003) and median PFS of 7 months (95% CI 5.1–8.9, *p* = .013) in the heterogeneous comparison group (Figure S2). Detailed survival data of all second‐line options are presented in Table S5.

## 4 DISCUSSION

Ipilimumab and nivolumab combination for patients with aRCC has emerged as one of the first‐line treatments of choice concerning efficacy and safety. The recently published 9‐year follow‐up of the CheckMate 214 trial confirmed the superiority of ipilimumab and nivolumab over sunitinib in both PFS and OS. Although IO combinations with TKI or a VEGF inhibitor show high response rates, ipilimumab and nivolumab are associated with a notably extended DoR to therapy.^3^, ^5^ IO/TKI‐regimes are often used in cases when rapid therapy response is needed, as the tumor shrinkage and therapy response seems to be faster than seen in the IO/IO‐regime. However, long‐term response seems to be most pronounced with ipilimumab and nivolumab.^17^

The current multi‐center (*n* = 17) analysis of 356 patients treated with ipilimumab and nivolumab in 2015–2023 in Germany and Switzerland offers important insights into the real‐world, complementing the findings from CheckMate 214 although certain points remain disparate.

Our cohort, for example, showed slightly worse outcomes compared to the CheckMate 214 cohort.^14^ Although ORR was similar between our and the CheckMate 214 cohort with 37.4% and 39.5%, respectively, the rates of CR and PR were 8.7% and 28.7% in our patients, aligned closely with the study cohort with 12% and 27.5%. Regarding the PFS and OS there are certain differences between our cohort compared to the pivotal study. The real‐world data demonstrated a median PFS of 8 months, which is relevantly shorter than the 12.4 months in CheckMate 214. Also, the median OS was 39 months compared to 52.7 months in the pivotal study. These differences could be explained by several factors. First, patients in our cohort had a worse performance status and more severe comorbidities (i.e., autoimmune diseases, impaired kidney function, etc.), which would have led to an exclusion from the pivotal study in 37.1% of patients. This is also reflected in the relevantly worse median OS (23 months, 95% CI 11.5–34.5) and ORR (29.3%) of the ineligible patients compared with the intention‐to‐treat (ITT) population.

Furthermore, the number of patients in the poor risk group was higher in our cohort than in the CheckMate 214 cohort with 28.7% versus 21% leading to an inferior prognosis at baseline.^16^ Notably, the inclusion of non‐ccRCC subtypes in our study, such as chromophobe and collecting duct RCC, further limits the direct comparability of our data with the pivotal study. These subtypes are usually more aggressive and partially less sensitive to the IO‐therapy than pure ccRCC, which was the only histological subtype included in CheckMate 214. Of note, the investigator‐assessed PFS in CheckMate 214 was slightly lower at 9.7 months and approaching the real‐world PFS of 8 months.^15^ This could be interpreted as a more realistic and clinically relevant representation of disease progression.

As mentioned previously, CheckMate 214 only included patients with clear cell histology, the SUNNIFORECAST and CheckMate 920 trials investigated the superiority of ipilimumab and nivolumab over sunitinib in non‐ccRCC. As reported in the European Society for Medical Oncology (ESMO) 2024, the final SUNNIFORECAST median OS was 42.4 months, with a median PFS of 5.5 months and an ORR of 32.8%.^18^, ^19^ In contrast, CheckMate 920 demonstrated lower median OS (21.2 months), median PFS (3.7 months), and ORR (19.6%), which aligns more with our survival data with a median OS of 20 months (95% CI 8.6–31.4), median PFS of 4 months (95% CI 1.5–6.5) and a ORR of 31.9% in the non‐ccRCC cohort.^20^ The significant differences in OS can likely be attributed to differences in the inclusion criteria between the CheckMate 920 and SUNNIFORECAST study and our real‐world cohort.

Nevertheless, the biological heterogeneity within non‐ccRCC warrants a further subgroup analysis. In our cohort, patients with a sarcomatoid differentiation showed better survival rates with ipilimumab and nivolumab compared to papillary or chromophobe carcinomas, as mentioned above. The SUNNIFORECAST trial showed no significant differences in treatment response between papillary and non‐papillary RCC, as well as a multi‐center study from Japan that included 44 patients.^12^, ^19^ In each case, there was no further grouping into chromophobe or sarcomatoid tumors, for example. As far as has been published to date, the CheckMate 920 study did not divide the non‐ccRCC group into subgroups in terms of response.^20^ Furthermore, to our knowledge, there are no larger prospective or retrospective studies that have investigated efficacy of ipilimumab and nivolumab in separated histological subgroups of non‐ccRCC. Our cohort also consists of small subgroups of non‐ccRCC (2.2%–8.1%), so further real‐world data focusing on this heterogeneous group should be collected to identify subgroups that may and may not benefit from therapy with ipilimumab and nivolumab.

In our study cohort, 7.6% of patients received ipilimumab and nivolumab despite having a favorable IMDC risk score. This might have been due to a wrong classification at therapy initiation and a re‐classification when their data were analyzed retrospectively or an off‐label use, when no other therapy was available. Patients with favorable risk showed a lower ORR compared to the intermediate risk group, which is consistent with the final results of the pivotal trial (29.6% vs. 29.6%). In CheckMate 214, 12.8% of patients achieved a CR, that was often durable and allowed therapy‐free intervals and subsequent therapy, respectively.^14^, ^15^ In our cohort, only two patients with favorable risk achieved a CR (7.4%). Both CR patients achieved durable responses over 3 years and almost 6 years, respectively. The differences compared to the pivotal study and intermediate risk cohort are most likely explained by the small number of patients in both studies.

Cox regression analysis revealed certain predictors for impaired median OS and PFS in our cohort. Significantly shorter survival (OS and PFS) was observed in patients with poor ECOG performance status, non‐clear cell histology, and lymphonodal metastases. While metastases of the liver, brain, and adrenal glands had an expected negative effect on median OS, lung and bone metastases were not associated with an inferior response.

Recently conducted multi‐center retrospective studies, which included a reduced number of patients, a reduced performance status^21^ and the presence of bone metastases were associated with significantly shorter survival or earlier progression, respectively.^22^, ^23^, ^24^

In a real‐world analysis of ipilimumab and nivolumab in metastatic melanoma, the presence of brain metastases and a poor ECOG score were also associated with a higher risk of death. Consistent with our results, immunosuppression (mostly with corticosteroids) was not associated with a worse but even a better outcome.^25^ These discrepancies in the results highlight the importance of further investigations of possible predictors of therapy response.

Our retrospective analysis showed that prior nephrectomy or metastasectomy as part of an interdisciplinary approach could have had a positive effect on the median OS of patients. Ohba et al. demonstrated that patients who underwent cytoreductive nephrectomy and received ipilimumab and nivolumab had improved PFS and OS, albeit in a small number of patients.^24^ However, in the case of surgical intervention, clinical experience suggests that the metastatic burden is correspondingly low. This suggests that the data may be biased and a further distinction whether these procedures were performed for cytoreduction or due to symptoms or complications, respectively, could not be made.

About 55.3% of our patients received all four induction cycles of ipilimumab and nivolumab, with these patients showing significantly longer overall and PFS. These data underscore that induction therapy to stimulate the immune system via the PD‐(L)1 and CTLA‐4 pathway could have a significant impact on patient response to therapy. These effects were also observed in the CheckMate 067 study in metastatic melanoma. Here, the combination of ipilimumab and nivolumab was superior to the respective monotherapy. In a Canadian multi‐center retrospective analysis of 195 aRCC patients, those who received fewer than four induction cycles also showed significantly worse OS and PFS. However, the number of patients in this group was significantly lower than in our cohort (9.2% vs. 44.7%).^26^ Although Ishihara et al. showed rather protective effects of an interruption of IO/IO‐therapy with regard to PFS in a small patient cohort, our and other relevant data underline the importance of induction therapy.^27^

In terms of safety, AEs of any grade occurred in 76.4% of patients in our cohort compared to 94.1% in the pivotal study; AEs ≥ Grade 3 occurred in 35.4% of our patients versus 49% in the CheckMate 214 trial. This is probably due to a documentation bias as patients within clinical trials are observed more cautiously than the patients in the real‐world setting, where AEs are rather documented when leading to therapy discontinuation or severe impairment of the patients' health and quality of life. Furthermore, patients in the real‐world might report fewer AEs to avoid therapy delays or discontinuation (reporting bias), compared to patients in the study cohort, where a new drug is tested and therefore a certain skepticism concerning its safety might be higher. Overall, frequently reported AEs were similar in the pivotal study and our cohort, including fatigue, erythema, thyroiditis, and diarrhea. Although there was a higher incidence of liver toxicity in our cohort (19.1% vs. 15% [alanine aminotransferase [ALT]] or 15% [aspartate aminotransferase [AST]]), therapy discontinuation rates were similar between the real‐world and the pivotal study. Ipilimumab or nivolumab were discontinued in 24.7% or 26.6% of our patients, respectively, in contrast to the pivotal study, where 24% of patients canceled either ipilimumab or nivolumab.^15^

Patients with AEs ≥ Grade 3 showed a relevantly poorer ORR with a remarkable low CR of 2.5% versus 9.3% in the group without AEs ≥ Grade 3. Similarly, the multivariate analysis showed a higher risk of death, although univariate analysis and also Kaplan–Meier did not show a reduction in median OS and median PFS. In patients with severe side effects, the use of corticosteroids for immunosuppression and thus a reduction in the effectiveness of immunotherapy would be assumed to be causal. However, patients treated with high‐dose corticosteroids showed no elevated HR for death or disease progression in Cox regression analysis. Due to the inconsistency of the data, clinical relevance on this point cannot be assumed and further investigations should be awaited.

A subsequent therapy was given in 51.1% of the cases in our cohort, with 63.4% receiving cabozantinib. In contrast, 58% of the patients in the CheckMate 214 trial received a subsequent therapy which consisted mostly of sunitinib (25%) and cabozantinib (23%). This difference is likely explained by the fact that cabozantinib was not an established standard of care when the CheckMate 214 study was conducted. While the CABOSUN trial demonstrated a superiority of cabozantinib over sunitinib, it did so in the first‐line setting.^28^ Nevertheless, these results and broader clinical experience and expertise in handling this drug, likely contributed to the growing use of cabozantinib also in subsequent therapy lines.

On ESMO 2024, the final results of the CaboPoint phase II study were presented and supported the use of cabozantinib as a valuable second‐line option after IO/IO‐therapy.^29^ The results showed a median OS of 24.3 months under cabozantinib after ipilimumab and nivolumab. In a retrospective analysis from Navani et al., the median OS for patients receiving cabozantinib after ipilimumab and nivolumab was similar at 21.4 months.^30^ Our findings show a significantly longer OS of 38 (95% CI 20.7–55.3, *p* = .003) and median PFS of 15 months (8.2–21.9, *p* = .013) under second‐line therapy with cabozantinib compared to heterogeneous comparison group. The median PFS in our patient cohort is comparable to CaboPoint data and another retrospective analysis of cabozantinib in second‐line after ipilimumab and nivolumab.^31^

Cabozantinib is approved as a monotherapy in the second‐line for patients who received another TKI. Therefore, its use following ipilimumab and nivolumab would constitute an off‐label application. In clinical practice, as observed in our group, cabozantinib was most frequently used off‐label following ipilimumab and nivolumab treatment and reflected a perceived benefit. In such cases, it is imperative to inform patients about the off‐label use. Further investigation in the use of cabozantinib after IO/IO‐therapy is needed.

## 5 LIMITATIONS

The main limitation of this study is its retrospective design, which lacks the advantages of a randomized trial. Comparing retrospective data to a prospective study always inherits certain structural limitations. Additionally, in our cohort radiologists did not follow strictly the RECIST criteria. Also, our study included fewer patients than the CheckMate 214 trial (356 vs. 550 in the ITT population) and there were no standardized follow‐ups.

However, the collection and analysis of real‐world data is important, to reflect on the safety and efficacy of new treatments implemented in patients outside of clinical trials and to confirm the study's reproducibility in the real‐world setting.

## 6 CONCLUSION

To our knowledge this is the first European multi‐center analysis of real‐world data from patients receiving ipilimumab and nivolumab as first‐line treatment in aRCC. With our analysis we underline the efficacy and safety in this setting even in a real‐world cohort of patients frailer than those in the pivotal study. We observed poorer survival likely due to patients with more and severe comorbidities and a high proportion (25.8%) with non‐ccRCC in our cohort. We did not find a significant increase in AEs and report a similar ORR as seen in the CheckMate 214 study.

## AUTHOR CONTRIBUTIONS

**Hendrik Dinkel:** Conceptualization; data curation; formal analysis; investigation; methodology; software; project administration; validation; visualization; writing – original draft; writing – review and editing. **Linus Materna:** Conceptualization; data curation; formal analysis; investigation; methodology; software; validation; writing – original draft. **Ramona Stelmach:** Data curation; investigation; formal analysis; validation; writing – review and editing. **Stefanie Zschäbitz:** Data curation; investigation; validation; formal analysis; writing – review and editing. **Stephanie Neuberger:** Data curation; investigation; validation; formal analysis; writing – review and editing. **Can D. Aydogdu:** Data curation; investigation; validation; formal analysis; writing – review and editing. **Jozefina Casuscelli:** Data curation; investigation; validation; formal analysis; writing – review and editing. **Timo Egenolf:** Data curation; investigation; validation; formal analysis; writing – review and editing. **Matteo Silberg:** Data curation; investigation; validation; formal analysis; writing – review and editing. **Julie Steinestel:** Data curation; investigation; validation; formal analysis; writing – review and editing. **Arne Strauss:** Data curation; investigation; validation; formal analysis; writing – review and editing. **Florian Kirchhoff:** Data curation; investigation; validation; formal analysis; writing – review and editing. **Marit Ahrens:** Data curation; investigation; validation; formal analysis; writing – review and editing. **Pia Paffenholz:** Data curation; investigation; validation; formal analysis; writing – review and editing. **Richard Cathomas:** Data curation; investigation; validation; formal analysis; writing – review and editing. **Berna C. Özdemir:** Data curation; investigation; validation; formal analysis; writing – review and editing. **Christopher Gossler:** Data curation; investigation; validation; formal analysis; writing – review and editing. **Philipp Ivanyi:** Data curation; investigation; validation; formal analysis; writing – review and editing. **Marc Rehlinghaus:** Data curation; investigation; validation; formal analysis; writing – review and editing. **Thomas Hilser:** Data curation; investigation; validation; formal analysis; writing – review and editing. **Viktor Grünwald:** Conceptualization; data curation; investigation; formal analysis; validation; methodology; writing – review and editing. **Katrin Schlack:** Conceptualization; data curation; formal analysis; investigation; methodology; project administration; software; supervision; validation; writing – review and editing.

## CONFLICT OF INTEREST STATEMENT

Hendrik Dinkel has received travel and accommodation support from Eisai. Linus Materna, did not declare any conflict of interest. Ramona Stelmach has served on advisory boards for Eisai, Amgen and Pfizer. She has received honoraria for lectures from Eisai and Pfizer and travel and accommodation support from Astellas, Ipsen, and Beigene. Stefanie Zschäbitz has received advisory board fees or honoraria (institutional and personal) from Amgen, Astellas, AstraZeneca, Bayer, Bristol‐Myers Squibb, Daiichi Sankyo, EUSA, Gilead, Ipsen, Johnson & Johnson, Merck, MSD, Novartis, Pfizer, Roche, Sanofi Aventis, StreamedUp, Urotrials, Urotube and Zentiva. Research funding was received from Eisai. Travel expenses were covered by Amgen, Astellas, AstraZeneca, Bayer, Eisai, Ipsen, Johnson & Johnson, Merck, MSD, and Pfizer. Stephanie Neuberger did not declare any conflict of interest. Can D. Aydogdu has received honoraria from Astellas and Ipsen. He has received travel support from Astellas, Bristol‐Myers Squibb, Eisai, Ipsen, Janssen, and MSD. Timo Egenolf received honorarium from Ipsen for a speaker engagement and participates in a peer‐to‐peer program by Merck. Matteo Silberg did not declare any conflict of interest. Julie Steinestel did not declare any conflict of interest. Florian Kirchhoff did not declare any conflict of interest. Marit Ahrens has received invitations to congresses from Bristol‐Myers Squibb, Deciphera, Eisai, IPSEN, Merck, Pfizer, and PharmaMar. She has participated in advisory boards for Apogepha, Boehringer Ingelheim, Bristol‐Myers Squibb, Deciphera, Eisai, EUSA Pharma, IPSEN, Janssen, Merck, MSD, Pfizer, and PharmaMar. She has delivered invited lectures for Streamed Up, FOMF and Onko Internetportal (DIGIMED). Pia Paffenholz did not declare any conflict of interest. Richard Cathomas has served in an advisory role on behalf of his institution for Astellas, AstraZeneca, Johnson & Johnson, MSD, Bristol‐Myers Squibb, Roche, Novartis, Pfizer and Ipsen. He has received institutional honoraria from Astellas, Johnson & Johnson, AstraZeneca, MSD and Roche. He declares no personal financial disclosures. Berna C. Özdemir received honoraria paid to her institution for lectures and advisory boards from Bristol‐Myers Squibb, MSD, Merck, Ipsen, Roche, Pfizer, Novartis, Janssen and Sanofi and participated in clinical trials sponsored by MSD, Bristol‐Myers Squibb, and Ipsen. Christopher Gossler did not declare any conflict of interest. Philipp Ivanyi reports employment or leadership positions as a membership on the Steering Board of the Immunooncology Cooperative Group (ICOG‐H), the Clinical Trial Steering Committee (CCC‐H), the IDMC Trial Adam (advisory board) as well as being a member of the Leader Group Head and Neck Cancer AIO and spokesman of the Interdisciplinary Working Group Kidney Cancer (IAGN‐DKG). He has served in advisory roles or provided expert testimony for Astella, Bayer, Bristol‐Myers Squibb, ClinSol, Deciphera, Eisai, EMD‐Serono, EUSA, H5‐Oncology, Ipsen, Merck Serono (Global), Metaplan, MSD, Onkowissen, Pfizer and Roche. He owns stock in BB‐Biotech. Honoraria were received from AIM, Apogepha, AstraZeneca, Astellas, Bayer (Europe and Global), Bristol‐Myers Squibb, CORE2ED, Deciphera, DKG‐Onkoweb, Eisai, EUSA, FOMF, Id‐Institut, Ipsen (Europe), Merck Serono (Europe and Global), MSD, MedKom, MTE‐Academy, MedWiss, New Concept Oncology, Onkowissen, Pharma Mare, Pfizer, Roche, ThinkWired!, Schmitz‐Communikation, StreamedUP!, Solution Academy, and Vivantis. The author has received research funding from AIO, AstraZeneca, Bristol‐Myers Squibb, EUSA, Eisai, GlaxoSmithKline, Ipsen, Lilly, Merck, MSD, Niedersächsische Krebsgesellschaft, Novartis, Pfizer, Roche, Serono, Stiftung Immunonkologie, and Wilhelm Sander Stiftung. Additional financial relationships include travel grants or reimbursements from Bayer, BB‐Biotech, Bristol‐Myers Squibb, Deutsche Gesellschaft für Thoraxchirurgie, EUSA, Ipsen, Merck, and Pharma Mare. Immaterial conflicts of interest include a membership in the German Working Party Medical Oncology (AIO), the German Cancer Society, ASCO, ESMO and the Oncological Working Party Hannover (OAK) as well as serving as spokesman of the Interdisciplinary Working Party—Kidney Cancer (IAGN‐DKG) and member of the Leading Groups for GU Cancer and Head and Neck Cancer within AIO. Marc Rehlinghaus has received honoraria for lectures and moderations from AstraZeneca, Merck, Astellas, and Reuter. He has served on advisory boards for Merck, Ipsen, Janssen, Novartis, and Bayer. He has received travel expenses and congress fee support from Merck, Janssen and Ipsen. Thomas Hilser has received honoraria from Ipsen. Viktor Grünwald has received honoraria for lectures from Bristol‐Myers Squibb, Ipsen, Eisai, MSD, Merck KGaA, AstraZeneca, AAA/Novartis, Amgen, Johnson & Johnson, Teilx Pharmaceuticals, Gilead Sciences, and Roche. He has served on advisory boards for Bristol‐Myers Squibb, Pfizer, Novartis, MSD, Ipsen, Johnson & Johnson, Eisai, Debiopharm, Gilead Sciences, Oncorena, Synthekine, and Recordati. He has received travel support from Pfizer, Johnson & Johnson, Merck KGaA, Ipsen, and Amgen. Katrin Schlack has received honoraria for lectures, consulting activities and preparation of educational events, as well as reimbursement of attendance fees for scientific meetings, from AAA, Amgen, Apogepha, Astellas, AstraZeneca, Bayer, Bristol‐Myers Squibb, Eisai, EUSA Pharma, Fosanis, Ipsen, Janssen‐Cilag, Merck Healthcare, MSD, Novartis, Pfizer, Roche, and Sanofi. Arne Strauss has received financial support for clinical trials and honoraria for advisory roles from Astellas, AstraZeneca, Bayer, Bristol‐Myers Squibb, Ipsen, Merck, MSD, and Pfizer. Consulting fees were received from AstraZeneca, Bayer, Bristol‐Myers Squibb, Merck, MSD, and Pfizer. Jozefina Casuscelli has served as an advisor to Eisai, MSD, and Bristol‐Myers Squibb and has received travel grants from Johnson & Johnson and Merck.

## ETHICS STATEMENT

The study was approved by each local ethical committee and was performed according to the guidelines of Good Clinical Practice. The Helsinki Declaration of 1975 (as revised in 2013) was complied with.

## Supporting information


**Data S1.** Supporting Information.

## Data Availability

The data supporting the findings of this study are provided in Supplementary Table 6. Other data and further information are available from the corresponding author upon reasonable request. [Correction added on 07 February 2026, after first online publication: The data availability statement has been updated.].
