# Supplementary material for: Real‐world efficacy and toxicity of ipilimumab and nivolumab as a first‐line treatment for advanced renal cell carcinoma according to IMDC risk criteria—A multi‐center retrospective analysis on behalf of the GUARDIANS group
Source: Int J Cancer. 2025 Nov 29;158(9):2440–51. doi: 10.1002/ijc.70267 (PMC12963715; doi:10.1002/ijc.70267)
Supplement: Supplementary file 1 — Data S1. Supporting Information. [file IJC-158-2440-s001.pdf]

Real-world efficacy and toxicity of ipilimumab and nivolumab as a first-line treatment for advanced renal cell carcinoma according to IMDC risk criteria - a multicenter retrospective analysis on behalf of the GUARDIANS Group

Hendrik Dinkel, Linus Materna, Ramona Stelmach, Stefanie Zschäbitz, Stephanie Neuberger, Can D. Aydogdu, Jozefina Casuscelli, Timo Egenolf, Matteo Silberg, Julie Steinestel, Arne Strauss, Florian Kirchhoff, Marit Ahrens, Pia Paffenholz, Richard Cathomas, Berna C. Özdemir, Christopher Gossler, Philipp Ivanyi, Marc Rehlinghaus, Thomas Hilser, Viktor Grünwald and Katrin Schlack

Table of Contents

Supplementary Table 1	Survival outcomes of aRCC patients with different histological subtypes treated with ipilimumab and nivolumab
Supplementary Table 2	Multi- and univariate analysis for OS of aRCC patients treated with ipilimumab and nivolumab
Supplementary Table 3	Multi- and univariate analysis for PFS of aRCC patients treated with ipilimumab and nivolumab
Supplementary Table 4	Median overall and progression-free survival of patients with and without AEs of any grade or AEs � grade 3
Supplementary Table 5	Survival outcomes of different second-line therapies following progression on ipilimumab and nivolumab treatment
Supplementary Table 6	Per patient level data collection points
Supplementary Figure 1	Kaplan-Meier estimates of OS (A) and PFS (B) in patients who received one, two, three or four cycles of ipilimumab and nivolumab induction therapy
Supplementary Figure 2	Kaplan-Meier estimates of OS (A) and PFS (B) in patients receiving second-line cabozantinib vs. other second-line options after ipilimumab and nivolumab

Supplementary Table 1: Survival outcomes of aRCC patients with different histological subtypes treated with ipilimumab and nivolumab
CI, confidence interval; IO, immuno-oncology; NR, not reached; OS, overall survival; PFS, progression free survival.

	**Median OS**	**Median PFS**
**Histological subtype (%)**	months (95 % CI)	months (95 % CI)
**Clear cell or predominant clear cell component (74.1)**	50 (NR-NR)	10 (6.7-13.3)
**Papillary (8.1)**	20 (0.0-43.5)	5 (1.8-8.2)
**Sarcomatoid (7.3)**	31 (NR-NR)	14 (5.9-22.1)
**Chromophobe (2.2)**	14 (8.9-19.1)	3 (1.7-4.3)
**Others or histology not known (8.2)**	11 (5.2-16.9)	3 (1.7-4.3)

Supplementary Table 2: Multi- and univariate analysis for OS of aRCC patients treated with ipilimumab and nivolumab
AE, adverse event; CI, confidence interval; ECOG, Eastern Cooperative Oncology Group Performance Status; HR, hazard ratio; No., number.

	Multivariate analysis		Univariate analysis	
**Variable**	HR (95 % CI)	p-value	HR (95 % CI)	p-value
**Age >65 years**<br>No<br>Yes	 1 (reference)<br>0.955 (0.616-1.48)	 0.836	 1 (reference)<br>0.932 (0.666 -1.304)	 0.681
**ECOG PS**<br>0-1<br>≥2	 1 (reference)<br>3.379 (1.86-6.138)	 **< 0.001**	 1 (reference)<br>3.649 (2.474-5.382)	 **< 0.001**
**Histology**<br>clear cell<br>non-clear cell	 1 (reference)<br>1.422 (0.84-2.407)	 0.19	 1 (reference)<br>1.782 (1.204-2.638)	 **0.004**
**No. of metastases**<br>1<br>≥2	 1 (reference)<br>0.543 (0.287-1.028)	 0.61	 1 (reference)<br>1.563 (1.019-2.397)	 **0.041**
**Lymph nodes**<br>No<br>Yes	 1 (reference)<br>1.293 (0.781-2.14)	 0.319	 1 (reference)<br>1.585 (1.122-2.238)	 **0.009**
**Lung**<br>No<br>Yes	 1 (reference)<br>1.47 (0.884-2.444)	 0.138	 1 (reference)<br>1.268 (0.866-1.857)	 0.223
**Liver**<br>No<br>Yes	 1 (reference)<br>2.343 (1.402-3.914)	 **0.001**	 1 (reference)<br>1.707 (1.167-2.499)	 **0.006**
**Bone**<br>No<br>Yes	 1 (reference)<br>1.233 (0.767-1.984)	 0.387	 1 (reference)<br>1.358 (0.969-1.904)	 0.075
**Brain**<br>No<br>Yes	 1 (reference)<br>1.703 (0.864-3.356)	 0.124	 1 (reference)<br>1.81 (1.102-2.974)	 **0.019**
**Adrenal glands**<br>No<br>Yes	 1 (reference)<br>1.374 (0.781-2.418)	 0.27	 1 (reference)<br>1.889 (1.258-2.837)	 **0.002**

**Prior nephrectomy**				
No	1 (reference)	**0.01**	1 (reference)	**<0.001**
Yes	0.506 (0.303-0.848)		0.334 (0.238-0.469)	
**Prior resection of metastases**				
No	1 (reference)	0.433	1 (reference)	**0.023**
Yes	0.814 (0.488-1.36)		0.616 (0.405-0.935)	
**AE ≥ grade 3**				
No	1 (reference)	**0.013**	1 (reference)	0.212
Yes	2.021 (1.158-3.529)		1.246 (0.882-1.762)	
**High dose steroids**				
No	1 (reference)	0.591	1 (reference)	0.846
Yes	0.857 (0.487-1.506)		1.041 (0.695-1.559)	

Supplementary Table 3: Multi- and univariate analysis for PFS of aRCC patients treated with ipilimumab and nivolumab

AE, adverse event; CI, confidence interval; ECOG, Eastern Cooperative Oncology Group Performance Status; HR, hazard ratio; No., number.

	Multivariate analysis		Univariate analysis	
**Variable**	HR (95 % CI)	p value	HR (95 % CI)	p value
**Age >65 years**<br>No<br>Yes	 1 (reference)<br>1.521 (1.098-2.108)	**0.012**	 1 (reference)<br>1.393 (1.069-1.814)	**0.014**
**ECOG**<br><2<br>≥2	 1 (reference)<br>1.492 (0.903-2.464)	0.118	 1 (reference)<br>1.216 (0.832-1.776)	0.313
**Histology**<br>clear cell<br>non-clear cell	 1 (reference)<br>1.349 (0.914-1.988)	0.13	 1 (reference)<br>1.593 (1.16-2.186)	**0.004**
**Nr. of metastases**<br>1<br>≥2	 1 (reference)<br>0.652 (0.409-1.039)	0.072	 1 (reference)<br>1.061 (0.78-1.441)	0.707
**Lymph nodes**<br>No<br>Yes	 1 (reference)<br>1.411 (0.97-2.053)	0.072	 1 (reference)<br>1.501 (1.15-1.959)	**0.003**
**Lung**<br>No<br>Yes	 1 (reference)<br>0.882 (0.613-1.27)	0.501	 1 (reference)<br>0.779 (0.589-1.030)	0.08
**Liver**<br>No<br>Yes	 1 (reference)<br>1.6 (1.046-2.449)	**0.03**	 1 (reference)<br>1.35 (0.987-1.845)	0.06
**Bone**<br>No<br>Yes	 1 (reference)<br>1.105 (0.781-1.563)	0.573	 1 (reference)<br>1.173 (0.896-1.534)	0.245
**Brain**<br>No<br>Yes	 1 (reference)<br>1.283 (0.715-2.302)	0.403	 1 (reference)<br>1.024 (0.632-1.658)	0.923
**Adrenal glands**<br>No<br>Yes	 1 (reference)<br>1.372 (0.865-2.177)	0.179	 1 (reference)<br>1.316 (0.92-1.88)	0.132

**Prior nephrectomy**<br>No<br>Yes	1 (reference)<br>0.922 (0.626-1.359)	0.682	1 (reference)<br>0.79 (0.599-1.043)	0.096
**Prior resection of metastases**<br>No<br>Yes	1 (reference)<br>1.071 (0.746-1.538)	0.709	1 (reference)<br>1.016 (0.761-1.357)	0.913
**AE ≥ grade 3**<br>No<br>Yes	1 (reference)<br>1.252 (0.846-1.853)	0.262	1 (reference)<br>0.941 (0.713-1.241)	0.666
**High dose steroids**<br>No<br>Yes	1 (reference)<br>0.757 (0.499-1.148)	0.19	1 (reference)<br>0.845 (0.613-1.165)	0.304

Supplementary Table 4: Median overall and progression-free survival of patients with and without AEs of any grade or AEs ≥ grade 3
AE, adverse event; CI, confidence interval; OS, overall survival; PFS, progression free survival

	Median OS		Median PFS	
	**months (95 % CI)**	**p value**	**months (95 % CI)**	**p value**
**AEs any grade**				
No	31 (13.5-48.5)	0.558	9 (3.68-14.32)	0.656
Yes	44 (31.4-56.6)		8 (5.7-10.3)	
**AEs ≥ grade 3**				
No	43 (27.4-58.6)	0.275	10 (6.5-13.3)	0.143
Yes	32 (16-48)		7 (5.4-8.6)	

Supplementary Table 5: Survival outcomes of different second-line therapies following progression on ipilimumab and nivolumab treatment

CI, confidence interval; IO, immuno-oncology; NR, not reached; OS, overall survival; PFS, progression free survival; TKI, tyrosine kinase inhibitor.

	Median OS	Median PFS
**Second-line therapy**	months (95 % CI)	months (95 % CI)
**Axitinib**	14.0 (3.7-24.3)	13.0 (2.7-23.3)
**Cabozantinib**	38.0 (20.7-55.3)	15.0 (8.2-21.8)
**Lenvatinib plus Everolimus**	NR (NR-NR)	NR (NR-NR)
**Pazopanib**	9.0 (NR-NR)	7.0 (2.8-11.2)
**Sunitinib**	16.0 (9.2-22.8)	7.0 (4.1-9.9)
**Tivozanib**	34.0 (NR-NR)	8.0 (4.8-11.2)
**Other (e.g. IO/TKI combination, belzutifan)**	18.0 (0-36.7)	11.0 (7.3-14.7)

Age at start of therapy	Age ≥ 65 years	Sex	ECOG performance status at start of therapy	Autoimmune disease	Relevant cardiovascular diseases	Chronic renal failure	Date of initial diagnosis of RCC	Date of initial diagnosis of advanced/metastatic RCC	Histology of RCC	Clear cell or clear cell component	Number of metastasis sites	LYM	PUL	HEP	OSS	BRA	ADR	PER	< 1 year from diagnosis to therapy start	KPS < 80%
years	*1=yes 2=no*	*1=female 2=male*	*0= ECOG 0, 1= ECOG 1, 2= ECOG 2, 3= ECOG 3, 4= ECOG 4*	*0=no 1=yes*	*0=no 1=CHD 2=heart failure 3= cardiac arrhythmia 4=thrombosis/ (pulmonary) embolism 5=other*	*0=no 1=GFR <60 ml/min 2=GFR <30 ml/min 3=dialysis required*	*DD.MM.YYYY*	*DD.MM.YYYY (if not primarily inoperable/metastatic)*	*1= clear cell, 2= papillary, 3= sarcomatoid, 4 = chromophobe, 5= other*	*1 = yes 2 = no*	*n*	*Lymph nodes 0=no 1=yes*	*Lungs 0=no 1=yes*	*Liver 0=no 1=yes*	*Bones 0=no 1=yes*	*Brain 0=no 1=yes*	*Adrenal gland 0=no 1=yes*	*Peritoneal 0=no 1=yes*	*0=no 1=yes*	*0=no 1=yes*
[illegible]	[illegible]	[illegible]	[illegible]	[illegible]	[illegible]	[illegible]	[illegible]	[illegible]	[illegible]	[illegible]	[illegible]	[illegible]	[illegible]	[illegible]	[illegible]	[illegible]	[illegible]	[illegible]	[illegible]	[illegible]

Hb < normal	Corrected serum calcium > normal	Neutrophils > normal	Thrombocytes > normal	IMDC score	Risk group (IMDC)	Nephrectomy/partial kidney resection	Metastasectomy	Lymph node dissection	Radiation	Ablation	Adjuvant immunotherapy	Date of start of therapy	Cycles of Ipi+Nivo received	Is treatment with nivolumab still ongoing?	If Nivolumab discontinued, date of end of treatment	Reason for discontinuation Nivo	1st staging	Response to treatment	Date of best response to treatment	Duration of response	Best response to treatment
0=no 1=yes	*0=no 1=yes*	*0=no 1=yes*	*0=no 1=yes*	*Total score*	*1=favorable risk (0 points), 2=intermediate risk (1-2 points), 3=poor risk (3-6 points)*	*0=no 1=yes*	*0=no 1=yes*	*0=no 1=yes*	*0=no 1=yes*	*0=no 1=yes*	*0=no 1=yes*	*DD.MM.YYYY*	*1=1 cycle 2=2 cycles 3=3 cycles 4=4 cycles*	*0=no 1=yes*	*DD.MM.YYYY*	*1=surgery/intervention 2=toxicity 3=PD 4=other*	*DD.MM.YYYY*	*1=CR, 2=PR, 3=SD, 4=PD, 5=mixed response, 6=staging pending 7=unknown*	*DD.MM.YYYY*	*months*	*1=CR, 2=PR, 3=SD, 4=PD, 5=mixed response, 6=staging pending 7=unknown*
[illegible]	[illegible]	[illegible]	[illegible]	[illegible]	[illegible]	[illegible]	[illegible]	[illegible]	[illegible]	[illegible]	[illegible]	[illegible]	[illegible]	[illegible]	[illegible]	[illegible]	[illegible]	[illegible]	[illegible]	[illegible]	[illegible]

Disease progression under therapy	If yes, date of progression	Progression-free survival	Follow-up therapy	Disease progression during follow-up therapy	PFS Follow-up therapy (progression until 2nd progression/last follow-up)	If yes, date of second progression (PFS2)	Follow-up after PD under follow-up therapy	Any irAE	Any irAE ≥ grade 3	Fatigue	Max. CTCAE fatigue	Weight loss	Max. CTCAE weight loss	Thyroiditis	Type of thyroiditis	Max. CTCAE thyroiditis	Hypophysitis	Max. CTCAE hypophysitis	Pancreatitis	Max. CTCAE Pancreatitis
0=no 1=yes	*DD.MM.YYYY*	*months*	*1=axitinib, 2=cabozantinib, 3=lenva/ever, 4=pazopanib, 5=sunitinib, 6=tivozanib 7=other*	*0=no 1=yes*	*months*	*DD.MM.YYYY*	*months*	*0=no 1=yes*	*0=no 1=yes*	*0=no 1=yes*	*1= Grade 1 2= Grade 2 3= Grade 3 4= Grade 4 5= Grade 5*	*0=no 1=yes*	*1= Grade 1 2= Grade 2 3= Grade 3 4= Grade 4 5= Grade 5*	*0=no 1=yes*	*1= Hypothyroidism 2= Hyperthyroidism 3= Both (sequential)*	*1= Grade 1 2= Grade 2 3= Grade 3 4= Grade 4 5= Grade 5*	*0=no 1=yes*	*1= Grade 1 2= Grade 2 3= Grade 3 4= Grade 4 5= Grade 5*	*0=no 1=yes*	*1= Grade 1 2= Grade 2 3= Grade 3 4= Grade 4 5= Grade 5*
[illegible]	[illegible]	[illegible]	[illegible]	[illegible]	[illegible]	[illegible]	[illegible]	[illegible]	[illegible]	[illegible]	[illegible]	[illegible]	[illegible]	[illegible]	[illegible]	[illegible]	[illegible]	[illegible]	[illegible]	[illegible]

Exanthema / rash	Max. CTCAE rash	Pruritus	Max. CTCAE Pruritus	Nausea	Max. CTCAE nausea	Diarrhea	Max. CTCAE diarrhea	Hepatitis / transaminase elevation	Max. CTCAE Hepatitis/transaminase elevation	Arthralgia	Max. CTCAE Arthralgia	Myalgia	Max. CTCAE Myalgia	Pneumonitis	Max. CTCAE Pneumonitis	Nephritis	Max. CTCAE nephritis	Need for corticosteroid therapy in irAEs?	High-dose corticosteroids (≥ 40 mg/d) received?	Last follow-up	Follow-up	Death	Cause of death
0=no 1=yes	*1= Grade 1 2= Grade 2 3= Grade 3 4= Grade 4 5= Grade 5*	*0=no 1=yes*	*1= Grade 1 2= Grade 2 3= Grade 3 4= Grade 4 5= Grade 5*	*0=no 1=yes*	*1= Grade 1 2= Grade 2 3= Grade 3 4= Grade 4 5= Grade 5*	*0=no 1=yes*	*1= Grade 1 2= Grade 2 3= Grade 3 4= Grade 4 5= Grade 5*	*0=no 1=yes*	*1= Grade 1 2= Grade 2 3= Grade 3 4= Grade 4 5= Grade 5*	*0=no 1=yes*	*1= Grade 1 2= Grade 2 3= Grade 3 4= Grade 4 5= Grade 5*	*0=no 1=yes*	*1= Grade 1 2= Grade 2 3= Grade 3 4= Grade 4 5= Grade 5*	*0=no 1=yes*	*1= Grade 1 2= Grade 2 3= Grade 3 4= Grade 4 5= Grade 5*	*0=no 1=yes*	*1= Grade 1 2= Grade 2 3= Grade 3 4= Grade 4 5= Grade 5*	*0=no, 1=yes*	*0=no 1=yes*	*DD.MM.YYYY*	*months*	*0=no 1=yes*	*1=disease progression 2=cardiac events 3=infection 4=other 5=unknown*
[illegible]	[illegible]	[illegible]	[illegible]	[illegible]	[illegible]	[illegible]	[illegible]	[illegible]	[illegible]	[illegible]	[illegible]	[illegible]	[illegible]	[illegible]	[illegible]	[illegible]	[illegible]	[illegible]	[illegible]	[illegible]	[illegible]	[illegible]	[illegible]

[illegible]

Supplementary Table 6: Retrospectively collected, coded and anonymized data of patients treated with ipilimumab and nivolumab.
AE, adverse event; CTCAE, Common Terminology Criteria for Adverse Events; ECOG, Eastern Cooperative Oncology Group Performance Status; Hb, Hemoglobin; IMDC, International Metastatic Renal-Cell Carcinoma Database Consortium Score; ir, immune-related; KPS, Karnowsky Performance Status; OS, overall survival; PFS, progression free survival; RCC, Renal Cell Carcinoma.

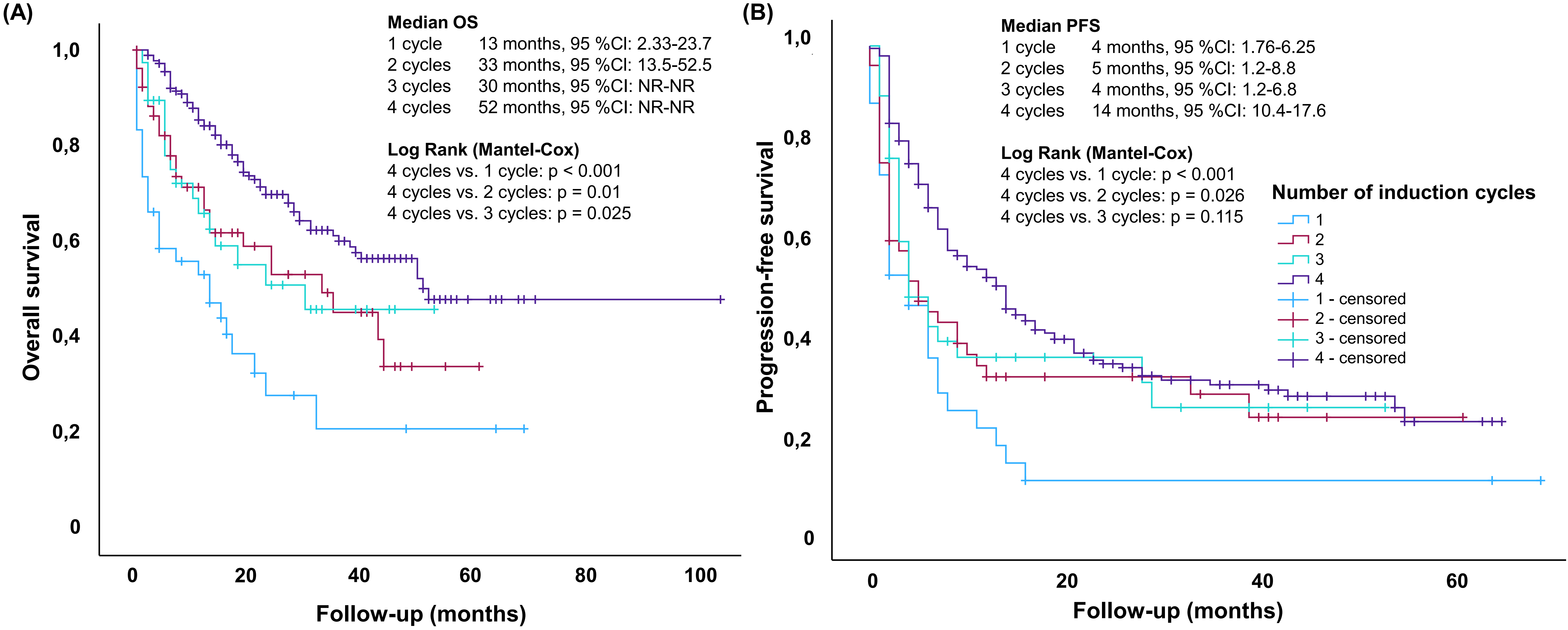

Time in months	0	3	6	12	18	24	30	36	42	48	54	60
**Patients at risk**												
1 cycle	41	26	21	18	8	5	3	2	2	2	1	1
2 cycles	51	42	36	28	22	18	14	10	8	2	1	0
3 cycles	37	32	26	20	13	11	9	5	3	0		
4 cycles	178	173	157	134	105	84	64	52	41	28	15	6
**cumulative events**												
1 cycle	1	14	18	19	25	26	27	27	27	27	27	27
2 cycles	1	7	11	16	18	21	21	23	23	25	25	25
3 cycles	1	4	9	12	15	16	17	17	17	17		
4 cycles	1	4	14	27	38	47	54	57	60	60	64	64

Time in months	0	3	6	12	18	24	30	36	42	48	54	60
**Patients at risk**												
1 cycle	30	16	10	5	1	1	1	1	1	1	1	1
2 cycles	48	28	21	13	10	9	8	5	2	0	0	0
3 cycles	38	21	14	10	8	6	4	3	1	0	0	
4 cycles	194	155	122	91	64	45	35	30	23	14	9	2
**cumulative events**												
1 cycle	4	17	21	26	28	28	28	28	28	28	28	28
2 cycles	2	21	27	33	33	33	34	35	35	35	35	35
3 cycles	1	15	21	23	23	24	25	25	25	25	25	
4 cycles	1	38	64	90	109	118	122	123	124	125	126	127

Supplementary Figure 1: Kaplan-Meier estimates of OS (A) and PFS (B) in patients who received one, two, three or four cycles of ipilimumab and nivolumab induction therapy

CI, confidence interval; NR, not reached; OS, overall survival; PFS, progression-free survival

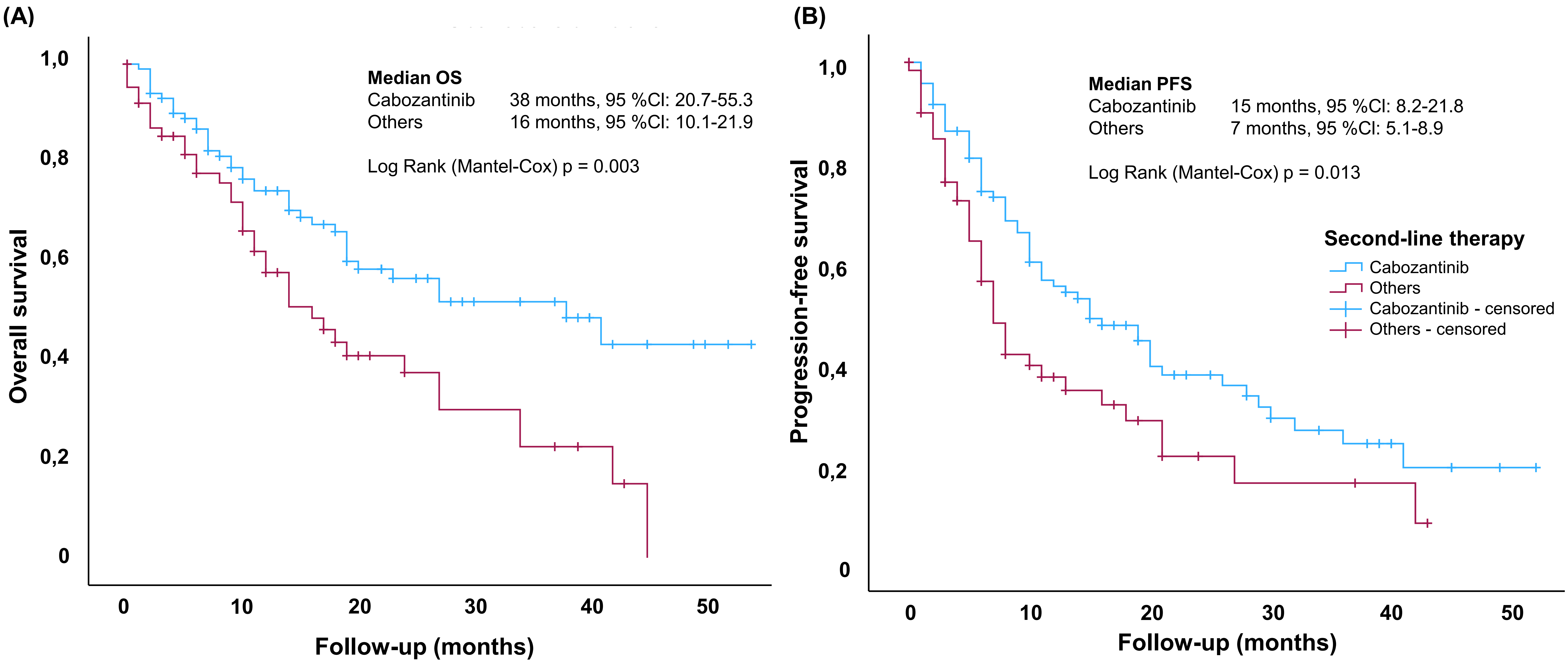

Time in months	0	3	6	12	18	24	30	36	42	48	54
Patients at risk											
Cabozantinib	101	92	79	60	44	27	19	16	7	3	0
Others	59	49	40	26	16	10	7	5	2		
cumulative events											
Cabozantinib	0	7	13	24	30	36	30	38	40	40	40
Others	3	9	13	23	29	31	34	35	42		

Time in months	0	3	6	12	18	24	30	36	42	48
Patients at risk										
Cabozantinib	95	81	65	46	31	18	12	9	3	1
Others	58	41	27	14	9	4	2	2	1	
cumulative events										
Cabozantinib	0	13	24	40	46	52	56	58	59	59
Others	1	14	24	33	36	38	39	39	40	

Supplementary Figure 2: Kaplan-Meier estimates of OS (A) and PFS (B) in patients receiving second-line cabozantinib vs. other second-line options after ipilimumab and nivolumab

AE, adverse event; CI, confidence interval; NR, not reached; OS, overall survival; PFS, progression-free survival
